# Supplementary material for: Surface acoustic wave hemolysis assay for evaluating stored red blood cells
Source: Lab Chip. 2025 Nov 6;26(1):40–53. doi: 10.1039/d5lc00652j (PMC12624846; doi:10.1039/d5lc00652j)
Supplement: LC-026-D5LC00652J-s001 [file LC-026-D5LC00652J-s001.pdf]

Supplementary Information (SI) for Lab on a Chip.
This journal is © The Royal Society of Chemistry 2025

Supporting Information for

Surface Acoustic Wave Hemolysis Assay for Evaluating Stored Red Blood Cells

Meiou Song[a, #], Colin C. Anderson[b, #], Nakul Sridhar[a], Julie A. Reisz[b], Leyla Akh[c], Yu Gao[a], Angelo D'Alessandro[b,d], Xiaoyun Ding[a,c,e,f]**

[a]Department of Mechanical Engineering, University of Colorado, Boulder, CO, 80309, USA.
[b]Department of Biochemistry and Molecular Genetics, University of Colorado Anschutz Medical Campus, Aurora, CO, 80045, USA.
[c]Biomedical Engineering Program, University of Colorado, Boulder, CO, 80309, USA.
[d]Omix Technologies Inc, Aurora, CO, 80045, USA
[e]*Material Science and Engineering Program, University of Colorado, Boulder, CO, 80309, USA.*
[f]*BioFrontiers Institute, University of Colorado, Boulder, CO 80309, USA.*

* Corresponding authors:
Xiaoyun.Ding@colorado.edu
ANGELO.DALESSANDRO@CUANSCHUTZ.EDU
#These authors contributed equally.

This PDF file includes:

Figures S1 to S8
Legends for Movies S1 to S2

Other supporting materials for this manuscript include the following:

Movies S1 to S2

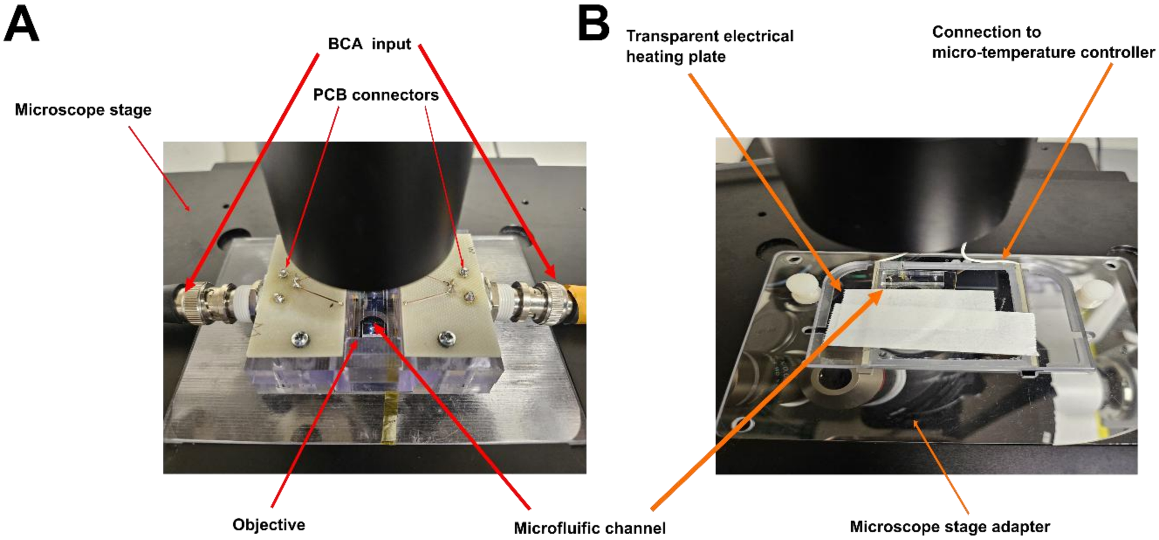

Fig. S1. Experimental setup of the SAW-HA devices and EHC. (A) SAW device mounted on a custom 3D-printed holder designed to fit securely onto the inverted microscope stage. RF signals are transmitted via BCA cables to PCB connectors, which deliver power to the IDTs on the SAW chip. (B) Transparent electrical heating plate with microscope stage adapter connected to micro-temperature controller for precise thermal control during EHC experiments.

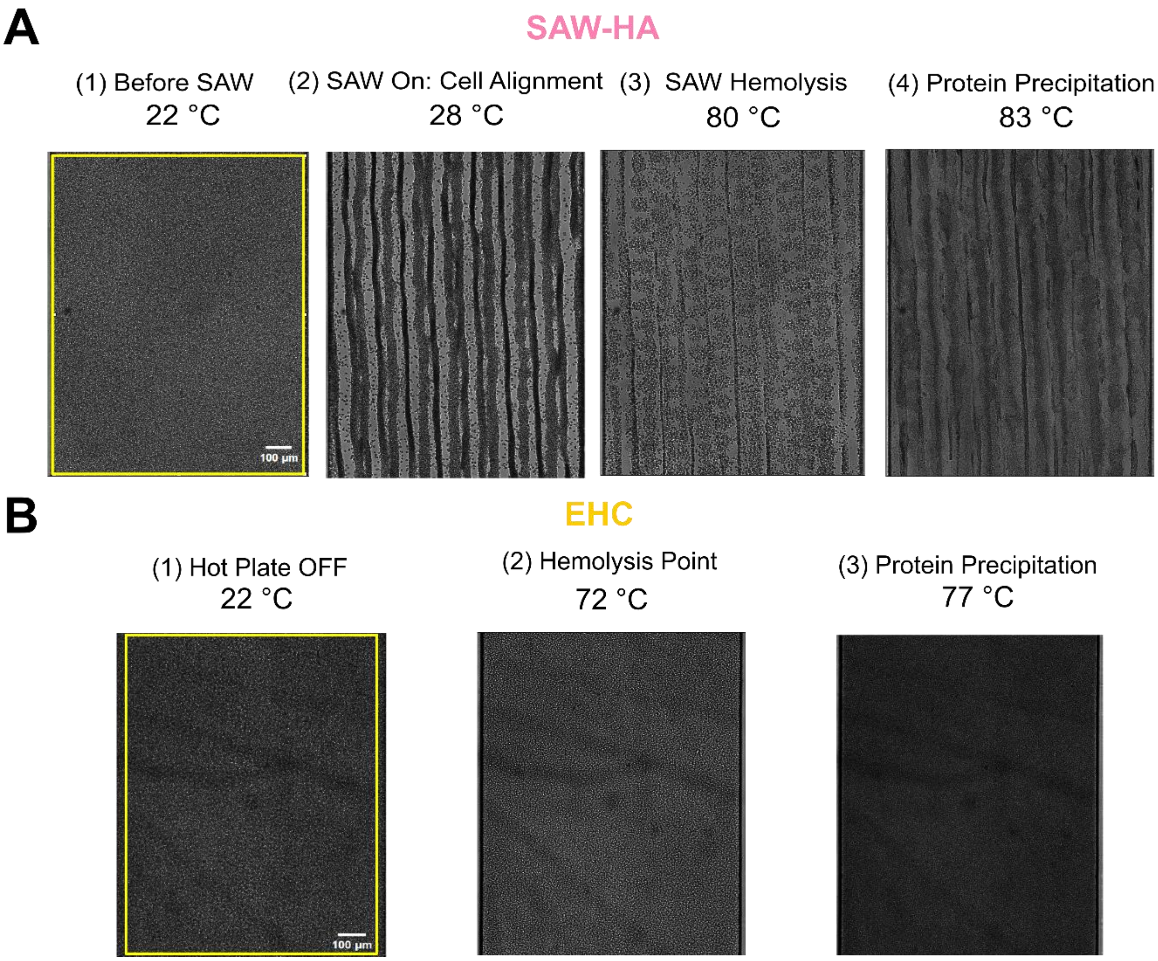

Fig. S2. Unaltered raw images for analysis of RBC behavior under SAW-HA and EHC. (A) Raw representative images showing RBC alignment to pressure nodes, RBC lysis, and protein aggregation under the SAW-HA. (B) Raw representative images showing RBC lysis and protein aggregation under EHC. Selected ROIs used for analysis (yellow rectangle: 1400 × 2000 pixels) are highlighted. Scale bar: 100 µm.

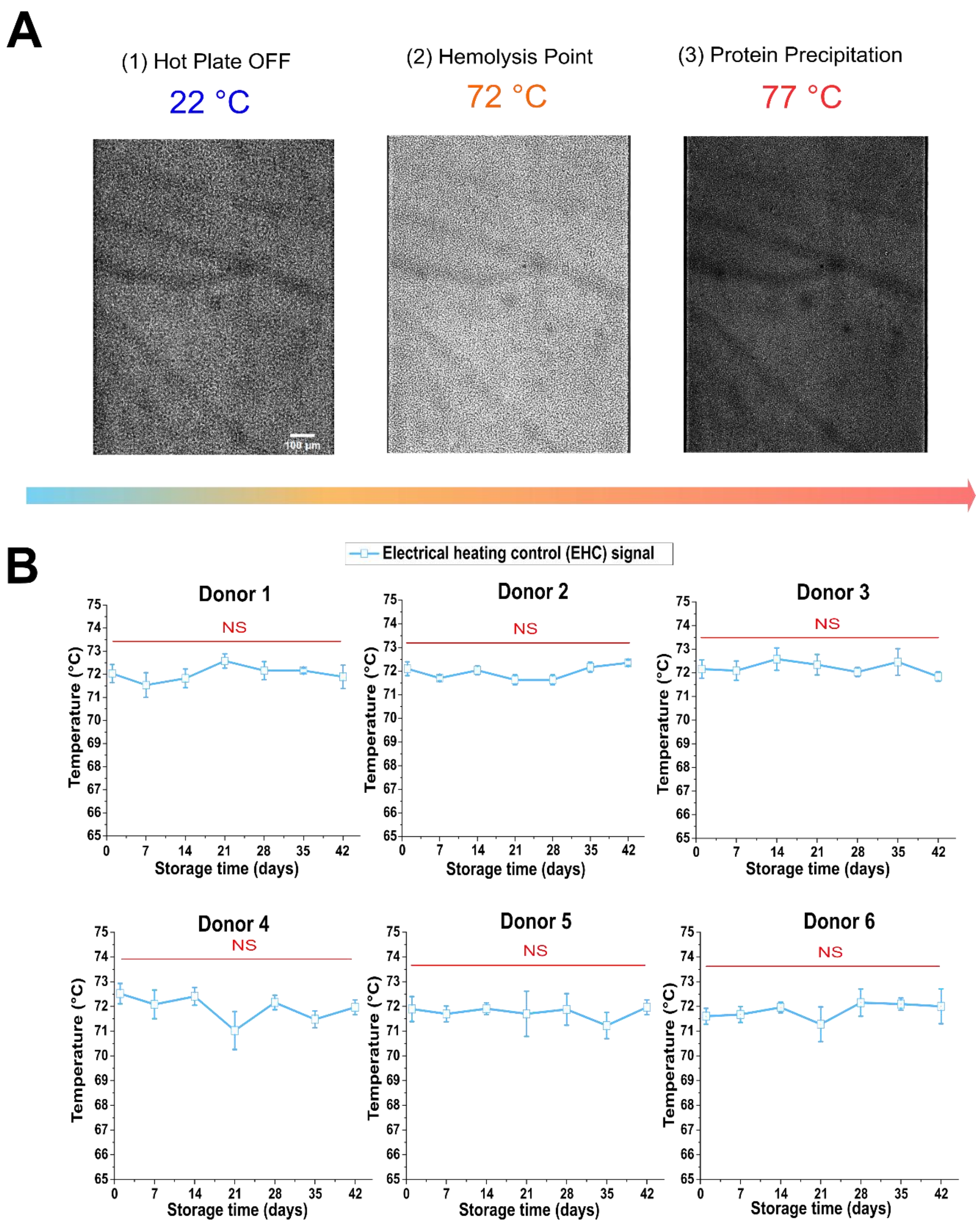

Fig. S3. RBC behavior under electrical heating control (EHC). (A) Enhanced contrast representative images showing key phases of RBC behavior under EHC. The images depict (1) the initial state of the RBC sample (22°C), (2) the hemolysis point (72°C), and (3) protein precipitation (77°C) as the sample is under electrical heating. Scale bar: 100 µm. (B) Weekly tracking of hemolysis temperatures for six biological samples (individual donors) during 42 days of cold storage under EHC. Each data point represents the mean of three technical replicates per donor per time point, shown with error bars (mean ± s.d.). The student t-tests of independence were performed in the above figures. Two-sided, unpaired t-test. NS, not significant.

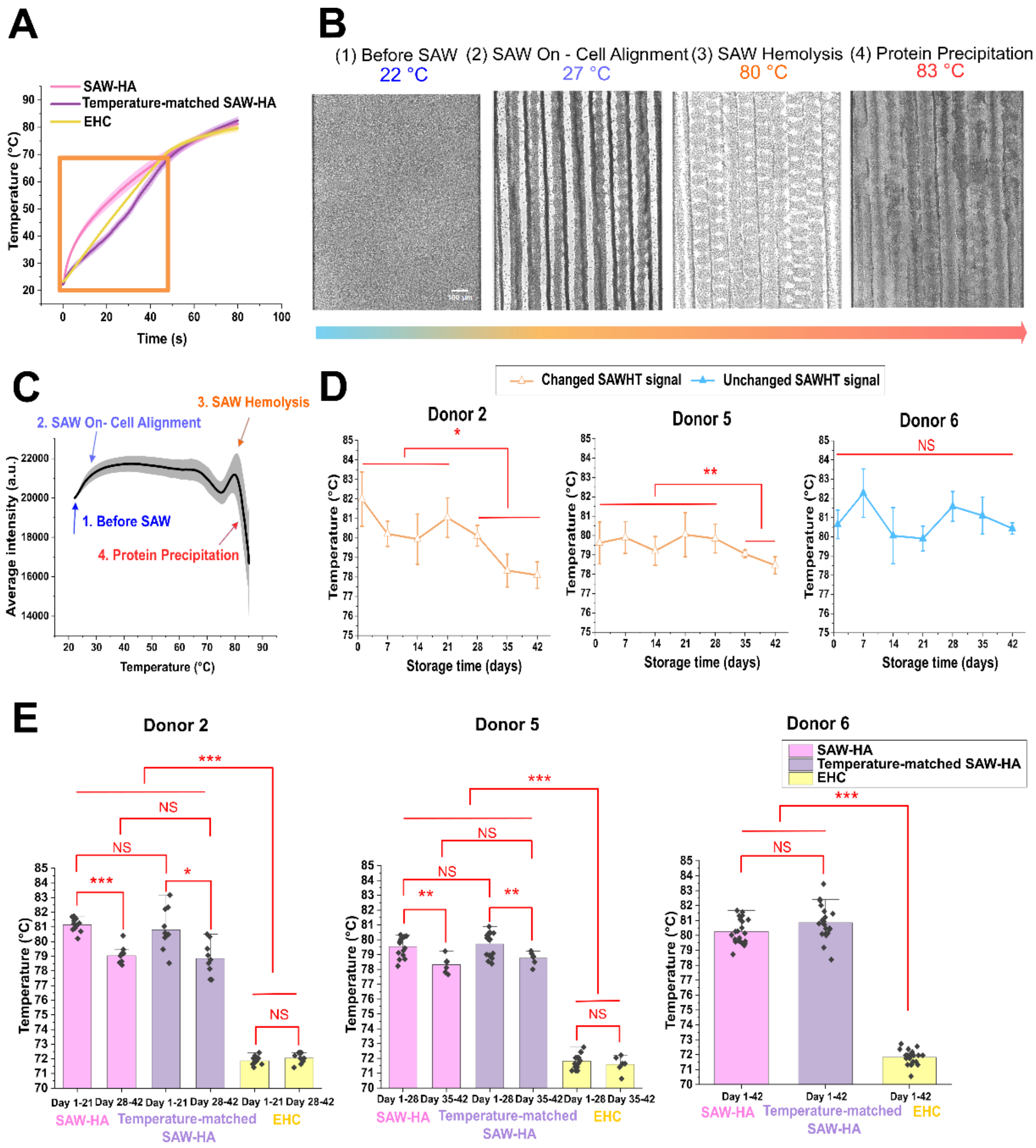

Fig. S4. Initial temperature differences do not contribute to acoustic signal shifts in measuring RBC storage quality changes. (A) Comparison of average temperature profiles among the SAW-HA, EHC, and a modified SAW-HA with an initial temperature profile matched to ETC (temperature-matched SAW-HA). The SAW-HA and EHC profiles represent averages from six donors (n=126), while the temperature-matched SAW-HA uses data from three donors (Donors 2, 5, and 6) (n=63). The orange square highlights differences in the initial temperature profiles among the methods. (B) Representative enhanced-contrast images showing key phases of RBC behavior (1: Before SAW, 2: SAW On – Cell Alignment, 3: SAW Hemolysis, 4: Protein Precipitation) with corresponding temperatures during the temperature-matched SAW-HA. Scale bar: 100 µm. (C) Average RBC lysis and protein precipitation curves generated by tracking grayscale intensity as a function of temperature using RBC samples from Donors 2, 5, and 6 (n=63). Key points in the curve correspond to the images in (B). (D) Weekly tracking of SAWHT for Donors 2, 5, and 6 over 42 days of cold storage using the temperature-matched SAW-HA. Data points represent means ± standard deviation from three technical replicates per week for each donor. (E) Comparison of weekly hemolysis temperatures among SAW-HA, EHC, and the temperature-matched SAW-HA for Donors 2, 5, and 6 (n=21 for each condition and for each donor). Statistical analysis was performed using a two-sided, unpaired Student's t-test. *P < 0.05, **P < 0.01, and ***P < 0.001; NS, not significant.

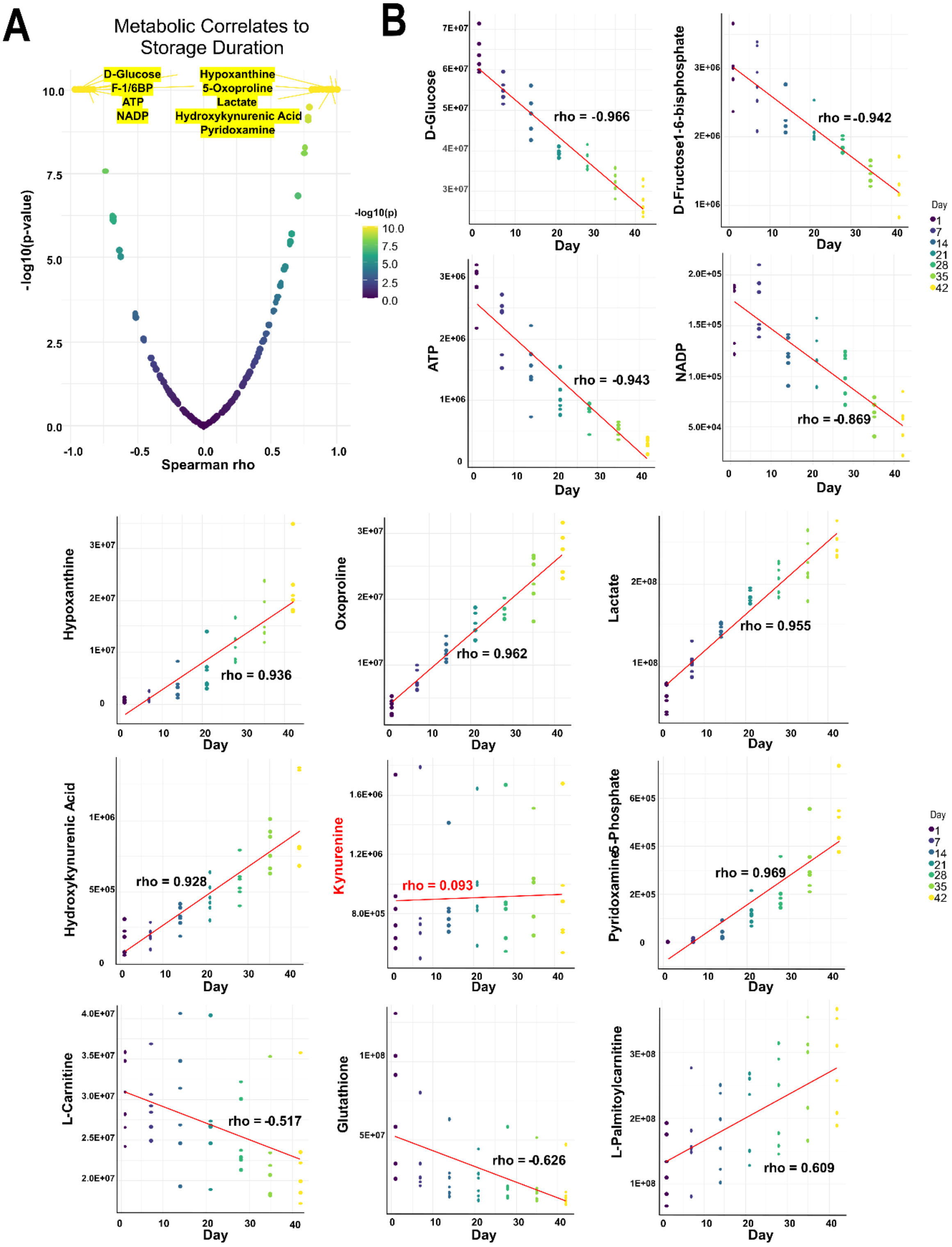

Fig. S5. All donor samples were evaluated for top metabolic correlates to storage duration. (A) A spearman correlation matrix was utilized to graph spearman rho vs p-value for metabolic correlates to storage duration with top correlates labeled (N = 42). (B) Select features were graphed over storage duration with correlations coefficient (N = 42).

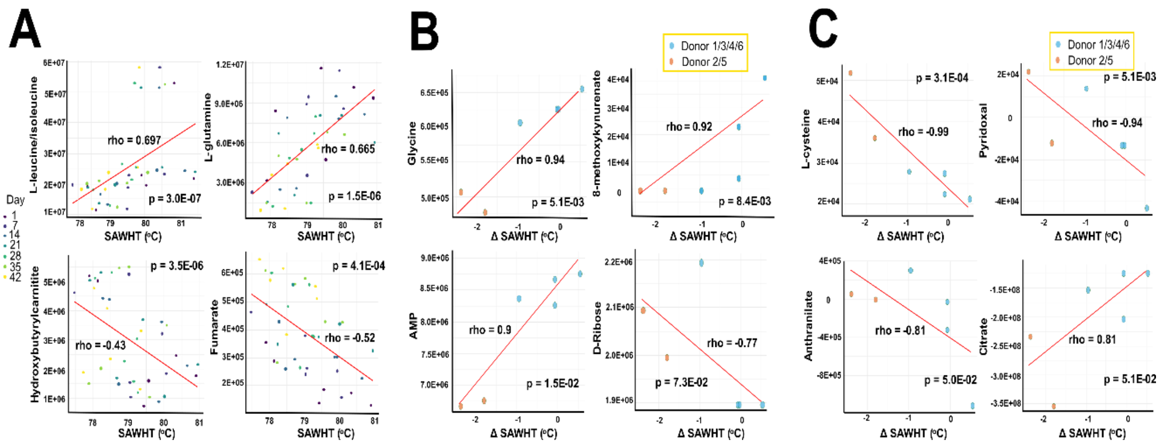

Fig. S6. Selected metabolite correlations with SAWHT. Individual metabolite scatter plots for top correlates identified in Fig. 4. (A) Selected correlates from Fig. 4A (metabolite abundance vs. SAWHT, N = 42). (B) Selected correlates from Fig. 4B (Day 1 abundance vs. ΔSAWHT, N = 6). (C) Selected correlates from Fig. 4C (change in abundance vs. ΔSAWHT, N = 6). For panels B and C, Donors 2 and 5 (changed acoustic signal group) are shown in orange; Donors 1, 3, 4, and 6 (unchanged acoustic signal group) are shown in light blue.

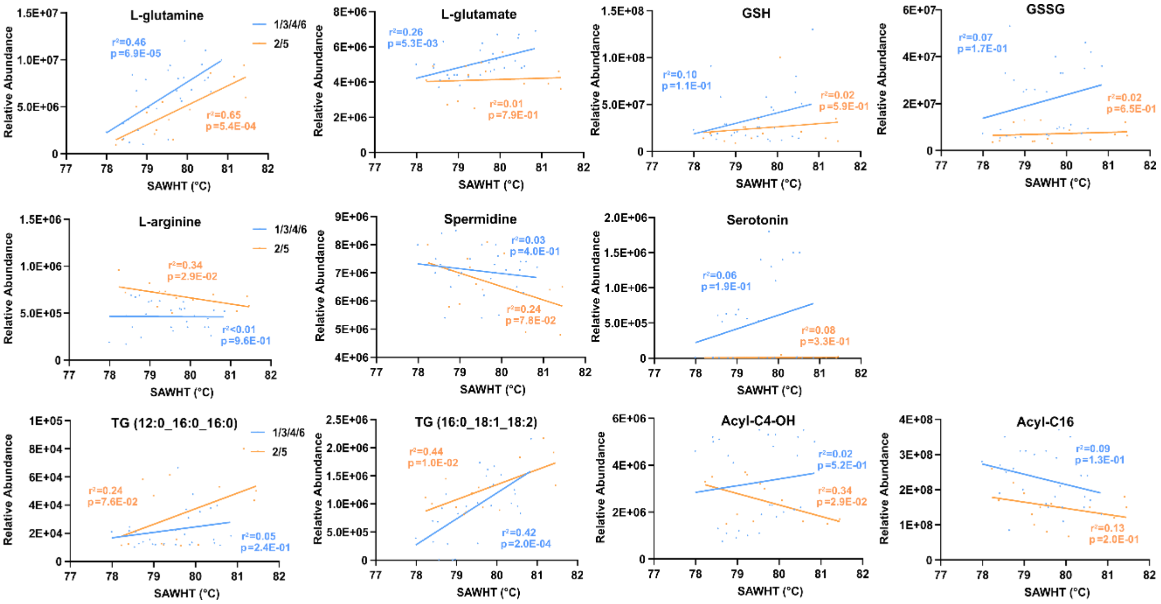

Fig. S7. Additional donor group-specific metabolite and lipid correlates of SAWHT. Extended set of selected metabolite and lipid scatter plots showing correlations between peak intensity (A.U.) and SAWHT for the two donor groups identified in Fig. 5. Donors 2 and 5 (changed acoustic signal group) (N = 14) are shown in orange; Donors 1, 3, 4, and 6 (unchanged acoustic signal group) (N=28) in light blue. Linear regression lines and correlation statistics are shown for each group.

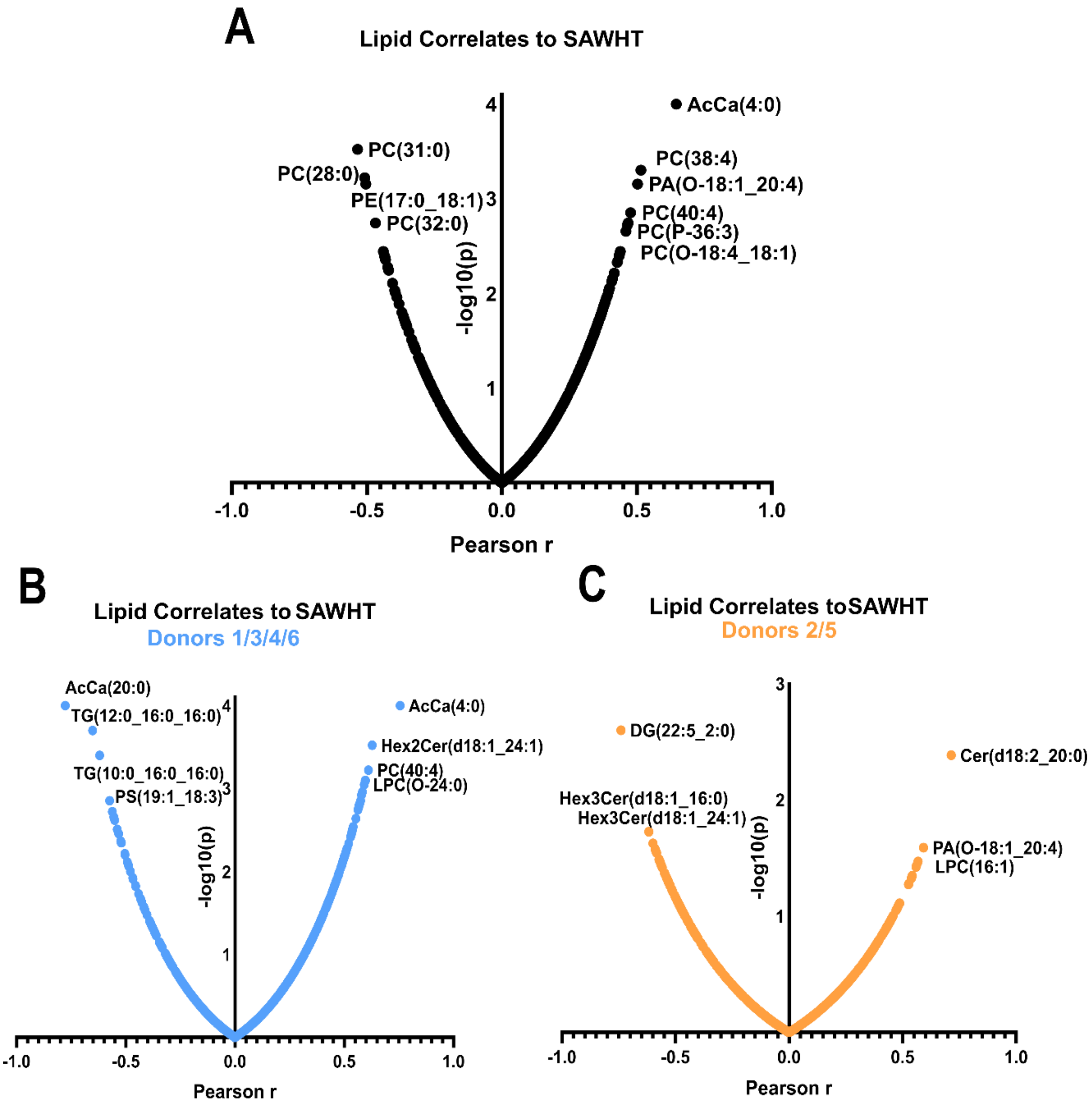

Fig. S8. A Pearson correlation analysis was performed between lipid peak intensity (A.U.) and SAWHT. Lipid correlates were graphed against p-value. Top lipid correlates are labeled for (A) All donors across all storage times versus SAWHT (N = 42), (B) Donors 1,3,4, and 6 (N = 28), and (C) Donors 2 and 5 (N = 14).

Movie S1 (separate file). SAW-HA application on an RBC sample. The video shows initial dispersed RBCs, immediate cell patterning upon standing SAW activation, followed by cell lysis and protein aggregation as the temperature in the microfluidic channel increases. The time scale in this movie has been compressed by a factor of ten.

Movie S2 (separate file). EHC application on an RBC sample. The video shows cell lysis and subsequent protein aggregation as the temperature increases under electrical heating. The time scale in this movie has been compressed by a factor of ten.
